# The role of three-dimensional in vitro models in modelling the inflammatory microenvironment associated with obesity in breast cancer

**DOI:** 10.1186/s13058-023-01700-w

**Published:** 2023-09-11

**Authors:** Rhianna Rachael Romany Blyth, Charles N. Birts, Stephen A. Beers

**Affiliations:** 1 Antibody and Vaccine Group, Centre for Cancer Immunology, School of Cancer Sciences, Faculty of Medicine, University of Southampton, Southampton, SO16 6YD UK; 2 School of Biological Sciences, Faculty of Environmental and Life Sciences, University of Southampton, Southampton, SO17 1BJ UK; 3 Institute for Life Sciences, University of Southampton, Southampton, SO17 1BJ UK

**Keywords:** Three-dimensional (3D) models, Breast cancer, Obesity, Adipose, Tumour microenvironment

## Abstract

Obesity is an established risk factor for breast cancer in postmenopausal women. However, the underlying biological mechanisms of how obesity contributes to breast cancer remains unclear. The inflammatory adipose microenvironment is central to breast cancer progression and has been shown to favour breast cancer cell growth and to reduce efficacy of anti-cancer treatments. Thus, it is imperative to further our understanding of the inflammatory microenvironment seen in breast cancer patients with obesity. Three-dimensional (3D) in vitro models offer a key tool in increasing our understanding of such complex interactions within the adipose microenvironment. This review discusses some of the approaches utilised to recapitulate the breast tumour microenvironment, including various co-culture and 3D in vitro models. We consider how these model systems contribute to the understanding of breast cancer research, with particular focus on the inflammatory tumour microenvironment. This review aims to provide insight and prospective future directions on the utility of such model systems for breast cancer research.

## Introduction

Breast cancer (BC) is the most common form of cancer among women globally, with an estimated annual 2.3 million cases worldwide [1]. Despite the increase in survival rates, it remains the second most common cause of mortality in women. The introduction of screening programs, improved understanding of disease pathogenesis, and greater utilisation of intervention therapies have all contributed to the continued reduction in BC-related mortality. However, there remains a growing incidence of BC globally, with current projections indicating that by 2030, worldwide cases will reach 2.7 million a year [2]. Thus, a greater understanding of BC development and progression, along with the models required to do this, is needed.

### Obesity and breast cancer

Approximately 23% of BC cases in the UK are avoidable due to lifestyle factors, with 8% of cases being caused by overweight and obesity [3]. Obesity is associated with an increased BC incidence and poorer survival outcomes. This is most established in postmenopausal women with oestrogen receptor (ER)-positive disease [4]. In the United States, the increased relative risk of breast cancer associated with excess body weight in postmenopausal women is 1.10 (1.08–1.12) per 5-unit increase in BMI [5]. However, there is increasing evidence that a high BMI is associated with poorer prognosis in BC patients of all ages [6, 7]. Conversely, BMI has been demonstrated to exhibit an inverse association with risk of premenopausal BC, though results from previous studies are inconsistent. Therefore, the underlying biological mechanisms of how obesity mediates BC remains unclear.

Interestingly, previous studies have shown that women classified as obese were also more likely to exhibit larger tumour sizes, lymph node involvement, higher propensity to distant metastasis, and lower distant disease-free interval, and overall survival [7–9]. However, this may be due to the issue of late-stage presentation, owing to the difficulty in performing clinical examinations (e.g. examination of larger breasts in women with obesity) and identifying tumours in overweight individuals [10].

In obese women, numerous local and systemic factors are hypothesised to support the link between breast cancer and obesity. Recent evidence highlights inflammation as a central mechanism through which obesity promotes cancer progression via effects in the local tumour microenvironment (TME), as well as systemic effects. In obesity, adipose tissue may promote breast cancer progression through the secretion of adipokines and inflammatory mediators [11]. Systemically, increased circulating levels of insulin and glucose, increased levels of oestrogens due to increased aromatase activity [12], insulin resistance [13], and hypercholesterolemia [14] have all been shown to contribute towards breast cancer development.

### The breast tumour microenvironment

The environment surrounding the tumour is referred to as the tumour microenvironment (TME) and can be divided into cellular, soluble, and physical components [15]. The cellular component can be further classified as intratumoral, regional (breast) or metastatic compartments. The intratumoral compartment refers to tumour cells and the tumour infiltrating cells such as lymphocytes, macrophages, and dendritic cells [16]. The regional compartment refers to adjacent stromal cells, including stromal fibroblasts, myoepithelial cells, and adipocytes [17]. The metastatic compartment refers to sites of metastases such as lymph nodes and distant organs [18]. The major cellular components of the breast TME are highlighted in Table 1. The crosstalk between BC cells and stromal cell populations as well as infiltrating immune cells induces phenotypic changes in the cellular components of the TME, resulting in extracellular matrix (ECM) remodelling and angiogenesis [19].

**Table 1:** Major factors within the breast tumour microenvironment

Factor	Function
Adipocytes	Adipocytes are a key source of metabolites, lipids and adipokines that can cause metabolic reprogramming of cancer cells, promoting proliferation, invasion, and resistance to therapy.
Cancer-associated fibroblasts (CAFs)	CAFs have a central role in regulating the tumour matrix. Heterogeneity of CAFs has been previously observed in BC, where subtypes have been shown to promote a cancer stem cell-like phenotype.
Myoepithelial cells	Epithelial cells that support luminal cells of the secretory mammary tissue. A loss of the intact myoepithelial ring surrounding BC cells shows a shift from non-invasive to invasive disease. However, myoepithelial cells have also been shown to drive suppression of BC.
Macrophages	Infiltration of macrophages into the adipose in obesity-associated BC leads to chronic inflammation and increased levels of pro-inflammatory macrophages. These macrophages form a ring around dying adipocytes called crown-like structures, which are associated with worse prognosis.
Extracellular matrix	The ECM has roles in cell adhesion, migration, and invasion. Matrix proteins such as fibrillar collagens, fibronectin and laminins are induced in breast cancer. Many of these ECM proteins play a role in breast tumour progression and metastasis. ECM remodelling enzymes become dysregulated in breast cancer, resulting in altered properties such as stiffness. In obesity, there is increased deposition of ECM and enhanced crosslinking of collagen fibres.

#### The adipose microenvironment

**Fig. 1:**
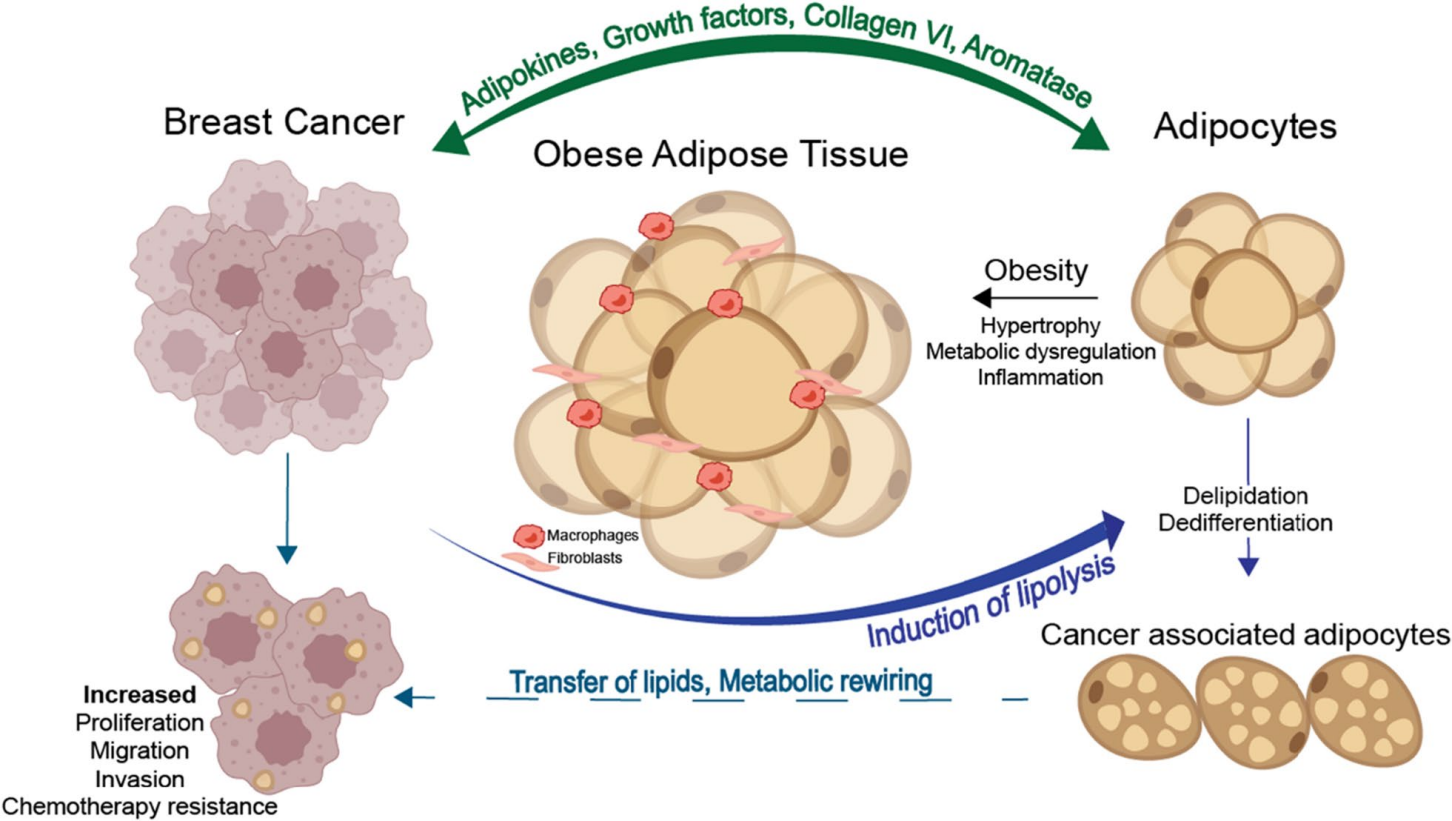
Crosstalk between adipose and breast cancer cells. Obesity leads to adipocyte hypertrophy, which induces the secretion of inflammatory adipokines, cytokines, and leptin. These adipokines induce polarisation and recruitment of macrophages. Macrophages secrete inflammatory cytokines, which can act on adipocytes to increase the expression of aromatase and oestrogen production and induce the expression of pro-angiogenic factors. Breast cancer cells reprogram adipocytes to induce delipidation and atrophy, transferring lipids to cancer cells, fuelling cancer cell growth, and resulting in increased proliferation, migration, and invasion. Original schematic created in Adobe Illustrator

The adipose microenvironment consists mainly of adipocytes alongside precursor adipose stem cells, fibroblasts, myofibroblasts, endothelial and immune cells. Adipocytes can adjust their number and morphology in response to energy balance via the processes involved in lipid uptake, lipolysis, and differentiation of pre-adipocytes [20]. During obesity, mature adipocytes expand in size and become hypertrophic, resulting in the secretion of inflammatory adipokines, growth factors and increased collagen production [21, 22]. The obese adipose microenvironment has been shown to favour cancer cell proliferation, promote angiogenesis and to reduce anti-cancer treatment efficacy (Fig. 1) [21, 22].

In an adipose-rich TME, cancer cells can alter the fate of adipocytes, forming cancer-associated adipocytes (CAAs) via adipocyte delipidation and atrophy [23, 24]. Reports have shown CAAs to be associated with increased production of pro-angiogenic and pro-inflammatory growth factors and cytokines such as IL-6, CXCL1 and TNF-α [25, 26], which can sustain angiogenesis and tumour progression [27]. Tumours may also benefit from CAAs as they provide fatty acids as an energy source to cancer cells. The transfer of lipids from adipocytes to cancer cells via lipolysis, has been previously reported in breast, ovarian and prostate cancer [24, 28, 29]. This lipolysis process results in the accumulation of fibroblast-like cells and a desmoplastic stroma, which suggests that some CAFs located at the adipose-tumour border might be derived from dedifferentiated adipocytes [23].

#### Obesity induced inflammation and crown-like structures

**Fig. 2:**
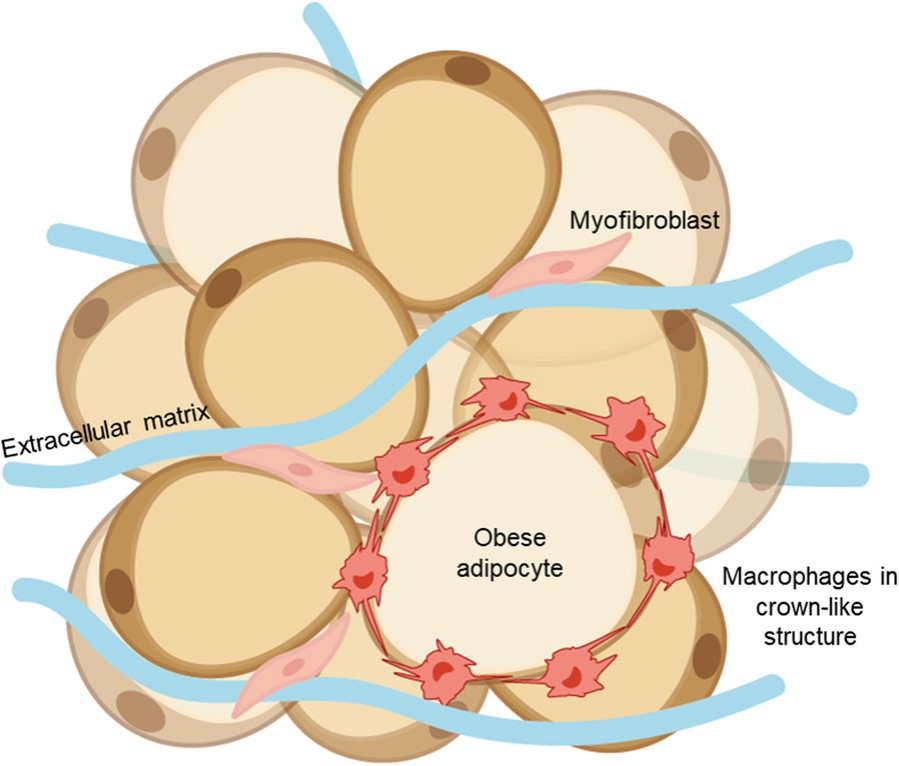
Formation and identification of crown-like structures. During obesity-associated inflammation, adipocytes become hypertrophic and enlarged. Pro-inflammatory macrophages form a ring around dying and necrotic adipocytes, forming a crown-like structure (CLS). Original schematic created in Adobe Illustrator

Macrophages contribute to the inflammatory state of the TME as one of the most abundant immune infiltrates [30, 31]. Greater infiltration of adipose tissue macrophages (ATMs) has been correlated with obesity, tumour size, recurrence, and development of tamoxifen resistance [32]. A characteristic of obesity is the presence of dying and necrotic adipocytes; identified by the presence of CD68-positive macrophages surrounding these dead cells, forming histological hallmarks known as crown-like structures (CLS) (Fig. 2) [33, 34]. CLS formation may result from increased levels of ATMs, contributing to both local and systemic inflammation. The presence of CLS is increased in breast AT in obese BC patients [12, 33]. However, CLS have also been reported in women with a normal BMI, who are classified as ‘metabolically obese’. For example, Iyengar and colleagues [12] identified an inflammatory state; characterised by the presence of CLS, associated with elevated aromatase levels, adipocyte hypertrophy and systemic metabolic dysfunction in women with a normal BMI. Therefore, it is still unknown whether the pro-inflammatory adipose microenvironment in obesity leads to increased CLS formation or vice versa.

Increased frequencies of CLS in individuals with obesity have been shown to negatively influence BC recurrence rates, survival, and therapy response [32, 35, 36]. CLS in obese AT has been shown to exhibit a mixed macrophage phenotype; characterised by the co-expression of surface markers that discriminate M1- such as CD11c, and M2-like macrophages such as CD206 or CD163 [37]. Furthermore, CLS localisation within the adipose microenvironment may play a role in macrophage phenotype. A recent study within our group showed that macrophages at the adipose-tumour border expressed both CD16 and CD32b, which may indicate a metabolically dysregulated macrophage phenotype [38].

CAAs and ATMs are central components of the BC niche. Nevertheless, their roles in the development of obesity-associated BC remains unclear. Understanding the mutual interaction between cancer cells, AT, and infiltrating immune cells could allow the possibility of overcoming AT-mediated therapy resistance and lead to novel therapeutic approaches.

## Modelling the breast tumour microenvironment

Most BC preclinical research is based on the use of BC cell lines as 2D monocultures. Traditional in vitro cell culture methods are generally less clinically relevant than preclinical models due to their inability to reflect the complex tissue architecture of tumours. Nonetheless, their low-cost, availability, scalability, and versatility make them invaluable tools in biomedical research for mechanistic, functional, and cellular profiling studies [39].

In vivo models play a central role in studying the cellular and molecular basis of BC progression. However, there are fundamental molecular and cellular differences between humans and mice, limiting the scope for animal models to fully recapitulate disease progression in humans. For instance, localisation of ER expression differs in the mammary glands of mice and humans [40]. Furthermore, differences between mice and humans have been highlighted in prior metastatic studies, where mice breast metastases often failed to colonise common sites of metastasis that occur in human BC such as the bone and brain [41]. However, this may be due to the route of tumour cell inoculation of the model system, such as the injection site, where metastases predominantly develop in the lungs.

A recent review showed that most novel anti-cancer drugs fail to enter the clinic due to limited efficacy or high toxicity [42]. This may in part be due to the lack of suitable models and overreliance of animal models. This has led to the development of alternative in vitro models to study human BC behaviour that maintain a 3D microenvironment. In accordance with the National Centre for the Replacement Refinement & Reduction of Animal Models in Research (NC3R’s) principles [43], there has been a vast development of 3D in vitro models.

**Fig. 3:**
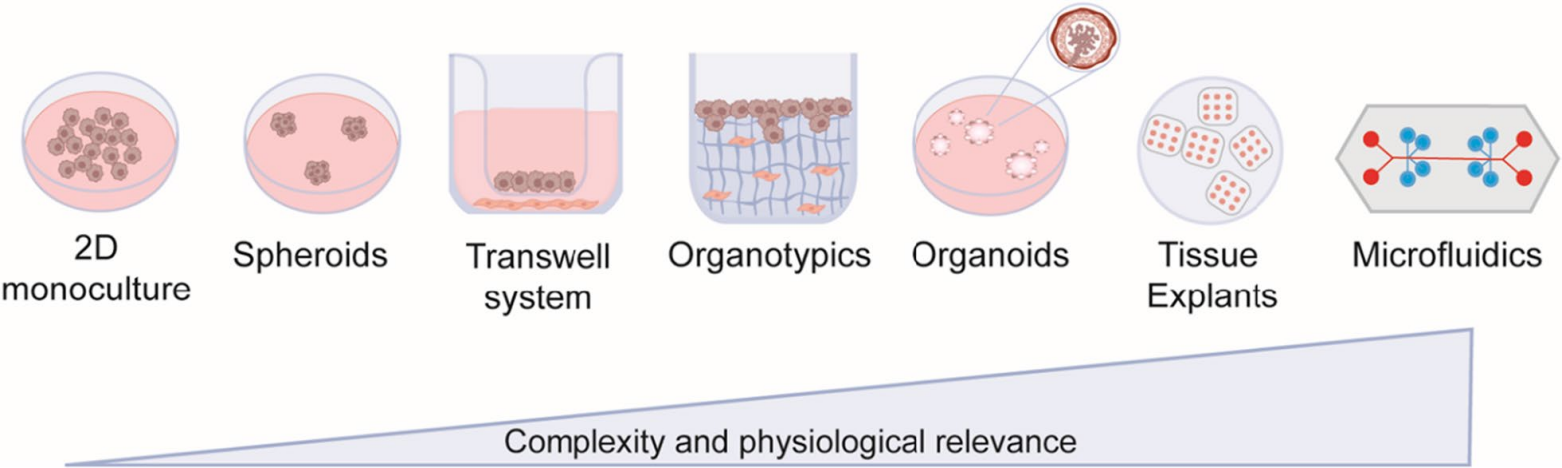
Breast cancer models for in vitro research. Traditional 2D cancer cell lines are easy to work with but lack tumour stroma, and adaptation to in vitro culture conditions results in loss of the original tumour phenotypic signature. Spheroids form from cellular aggregates which may be cultured from one or more cell types in suspension or within a hydrogel. Transwell systems involve the compartmentalisation of different cell populations, limited by the lack of direct cellular and cell-ECM interactions. 3D breast organotypic cancer models entail a specialised hydrogel approach in which cancer cells are seeded into a hydrogel laden with cell populations such as fibroblasts. Organotypic models are now widely used as they are a physiologically relevant system and closely resemble the original tumour. Co-culture systems containing fibroblasts, endothelial cells or immune cells have extended the predictive capabilities of these systems. Organoids self-assemble in multicellular structures which closely resemble the organisation of host tissue. Tissue explants are isolated tissue segments, capable of being cultured ex vivo, and retain physiological characteristics of the breast tumour. Microfluidic systems entail bespoke bioengineered chips facilitating fluid flow and recapitulating circulation. Original schematic created in Adobe Illustrator

Numerous 3D in vitro models have already been established, including spheroids, organoids, and microfluidic tumour-on-a-chip. Figure 3 demonstrates the most common 3D in vitro cultures currently used in BC research. Utilising 3D in vitro models to uncover cellular interactions and crosstalk within the TME will further understanding of BC progression. Furthermore, this could allow the possibility of identifying novel therapeutic approaches, improving current anti-cancer therapies and patient outcomes by specifically targeting the TME.

### Three-dimensional modelling of the obese breast TME

The complexity of 3D culture systems is heavily dependent on the cell types being introduced. For example, co-culture systems containing three or more cell types show greater complexity and physiological relevance than monoculture systems. However, culturing various cell populations within the same system may be challenging due to different favoured growth conditions.

Adipocytes account for the largest proportion of stromal cells in the breast. However, few studies have incorporated adipocytes into 3D models of BC, and this may be due to the challenges of culturing adipocytes. The culture and differentiation of pre-adipocytes into mature adipocytes requires specialist differentiation growth medium over an extended period. Additionally, the incorporation of adipocytes into a 3D model remains a challenge due to their increased buoyancy and inability to form a distinct cell pellet. Therefore, this review will outline the current approaches being developed to model the obese breast TME, focussing on the incorporation of macrophages and adipocytes into co-culture systems.

#### Spheroids

Spheroids represent the most used liquid-based 3D tumour culture system, capable of recreating tumour characteristics that are not seen with traditional 2D cultures, such as hypoxia [44, 45]. During spheroid development, cells adhere to each other to form a spherical cell mass allowing cell–cell interactions. Spheroids can be obtained from monocultures or co-cultures with stromal cells. Spheroids are routinely cultured in low attachment plates under matrix-free conditions. However, increasing studies have furthered this model by embedding spheroids in a permeable matrix (hydrogel), allowing for interactions with the ECM to be investigated.

##### Applications of spheroids in breast cancer research

In a recent study, 3D cultures of the ER + MCF-7 cell line, showed higher expression levels of the cancer stem cell-like marker CD44 when compared with their culture in 2D [46]. The difference in gene expression profiles of 2D vs 3D cultures could be a result of cell–cell and cell-ECM interactions generated by the TME and are involved in the development and progression of BC [47, 48].

Spheroid culture systems have been combined with scaffold-based methods, that utilise an artificial ECM to generate 3D co-cultures which better reflect cell-stroma interactions. Additionally, more recent approaches have incorporated immune cells such as macrophages to generate multicellular spheroids. Tevis et al*.* generated a TNBC ‘heterospheroid’, containing breast tumour cells and macrophages embedded in a collagen gel [49]. This model displayed increased secretion of IL-10, suggesting that the macrophages adopt a more M2-like phenotype upon co-culture with MDA-MB-231 cells. Furthermore, this model exhibited resistance to paclitaxel treatment in comparison with MDA-MB-231 monoculture spheroids. They also developed a second model whereby the macrophages were embedded in collagen surrounding the tumour spheroid. Despite the same cell populations used and the ratio of cells being consistent across both models, they exhibited distinct behaviours. Therefore, the way in which cell populations are incorporated into 3D models is important for modelling different aspects of BC such as stroma vs tumour.

Furthermore, in 2021, Horder et al*.,* generated a method of generating mature adipocyte spheroids from human adipose-derived stem cells (ADSC) [50]. Hydrogels containing BC cells were then seeded onto the adipocyte laden constructs. Co-cultures displayed a cancer cell-induced lipolysis and reduction of the adipocyte lipid content, characteristic of CAAs. Furthermore, ECM remodelling had occurred within the AT, exhibiting increased fibronectin, collagen I and collagen VI expression. This showed that spheroid co-culture systems could, at least in part, recapitulate central components of the complex cell–cell and cell-ECM interplay within the adipose microenvironment.

#### Hydrogels

Most 3D models utilise a porous, permeable ECM as their main component to model the TME. The ECM plays important roles in cell adhesion, migration, invasion, and signalling. Therefore, modelling the ECM allows for preservation of the molecular and phenotypic characteristics of these multicellular structures. Multiple cell types can be embedded into a hydrogel matrix, which is polymerised via incubation at 37°C and maintained in culture over days, up to weeks. The most common hydrogels are extracted matrix proteins such as rat-tail collagen and Matrigel®. However, synthetic hydrogels, produced with polymers such as polyethylene glycol (PEG), are increasingly being used as they allow for control over matrix properties [51]. Synthetic hydrogel components can be modified to mimic ECM properties of distinct TMEs. For example, a recent study developed an adipose-derived hydrogel that supports the adipogenic differentiation of adipose-derived stem cells (ADSC’s) [52].

Hydrogels offer a tuneable system, where stiffness and matrix proteins can be monitored. Once embedded in the matrix, cells have been shown to form physiologically relevant structures, remodel matrix, and interact with each other within a 3D environment. An example of this is the generation of breast duct-like structures. In 2017, Carter et al*.* embedded isolated populations of primary luminal and myoepithelial cells in a hydrogel matrix, where they formed duct-like structures in 3D [53]. Therefore, hydrogels are a versatile tool used to study cell–matrix and cell–cell interactions in a 3D environment, allowing for the formation of physiological multicellular structures.

However, ECM-derived hydrogels such as collagen type I and Matrigel® are disadvantaged by their variability, limited stiffness range and structural instability caused by hydrogel shrinkage and degradation during culture. Furthermore, recovery of cells from these hydrogels for down-stream applications requires proteolytic digestion that may negatively impact cells.

### Transwell and migration assays

Transwell migration and invasion assays utilise a two-chamber system (Boyden Chamber), where a membrane or ECM layer separates two cell populations. In principle, chemoattractants in the lower chamber can induce cell migration in the upper chamber, enabling the measure of invasion through the ECM. This system can be used to uncover mechanisms of invasion and metastasis. Transwell systems provide a low-cost easily implemented and high throughput assay, though these systems are not very physiologically relevant as they rely on culturing cells in 2D.

To investigate the role of adipocytes in BC progression, Jafari et al*.,* developed a transwell system to co-culture ER + BC cells with adipocytes obtained from cancer-free patients with or without type II diabetes (T2D). Adipocytes from insulin-resistant and T2D patients induced the expression of genes involved in EMT and increased BC cancer migration upon co-culture [54].

Cao et al. [55] utilised a transwell system to model immunometabolic pathways in BC associated with obesity. High leptin levels have been found to be associated with increased BC risk [56]. Leptin has been shown to induce macrophage recruitment and stimulate macrophage secretion of pro-inflammatory cytokines such as IL-6 and TNF-α [57, 58]. Co-culture of MCF-7 and MDA-MB-231 cells with leptin-treated M2 macrophages significantly induced BC migration and invasion [55]. Furthermore, treatment with a functional neutralising antibody against IL-8 significantly blocked invasion of MCF-7 and MDA-MB-231 cells. Therefore, this study demonstrated that IL-8 secretion from leptin-treated M2 macrophages may stimulate BC cell migration and invasion.

These models reflect a high throughput and low-cost measure of cancer cell migration. However, such systems do not account for cell-ECM interactions and therefore, hold low physiological relevance. To better replicate the in vivo microenvironment, 3D scaffolds have also been introduced in transwell-based co-culture systems.

Rebeaud et al*.*, developed a novel 3D culture system for human primary adipocytes from obese BC patients to study the metabolic crosstalk between BC cells and adipocytes in an obese environment [59]. Adipocytes isolated from primary mammary AT were embedded in a fibrin matrix, and co-cultured with BC cells in a transwell system. After 2 days, they observed the transfer of free fatty acids between adipocytes and BC cells. This study outlined the first protocol for culturing primary adipocytes for longer than 3 days, providing a new tool for investigating the obese breast TME.

### Organotypic models

Organotypic cultures typically utilise hydrogels which act to create a compartmentalised system. The hydrogel, which can be embedded with cell populations from the TME such as CAFs, is either submerged in culture media or raised to the air–liquid surface. These systems are typically cultured for days to weeks, allowing for the formation of complex interactions. Unfortunately, most organotypic cultures rely on end-point histopathological analysis, which reduces the 3D complex structures to a 2D ‘snapshot’ of cellular interactions and behaviour. However, fluorescent imaging and confocal microscopy have provided useful tools for capturing whole-gel structures and interactions.

These model systems have revealed mechanistic interactions between cancer cells and the TME. It has been reported that cancer cells that exhibit an epithelial phenotype such as luminal BC cells, cannot easily invade through acellular organotypic gels [39]. However, in the presence of CAFs, cancer cell invasion becomes apparent. CAFs can manipulate the organotypic ECM to favour cancer cell growth and invasion.

#### Applications of organotypic cultures

Organotypic models can be modified to incorporate multiple cell types and use different matrices. A study in 2009, developed a novel organotypic model of normal breast and the BC microenvironment [60]. This was the first study to report the co-culture of three major components of the normal and malignant breast: luminal cells, myoepithelial cells, and stromal fibroblasts in a collagen matrix. This model exhibited co-unit duct-like structures recapitulating normal and DCIS breast, where myoepithelial cells localised around the luminal population. Malignant co-units displayed disruption of the basement membrane and loss of β4-integrin, characteristic of DCIS. Interestingly, addition of normal fibroblasts did not influence co-unit formation. Whereas inclusion of CAFs resulted in disruption of co-unit organisation. These results support the value of these models in dissecting normal and tumour cell behaviour to uncover mechanisms of BC progression. However, this system involved the co-culture of three cell populations, which does not fully recapitulate the breast TME.

Recent studies have incorporated adipocytes into organotypic models to better reflect obesity associated BC. Bougaret et al*.* developed a co-culture model of ER + BC cells with mammary adipocytes isolated from women of normal weight, overweight and obese [22]. Through this model, they demonstrated that the anti-proliferative effects of tamoxifen were no longer seen when obese mammary adipocytes were present. Similarly, in another study, co-culturing MCF-7 cells with mammary adipocytes exposed to high glucose resulted in decreased tamoxifen responsiveness of BC cells [61]. However, sensitivity was regained by inhibiting adipocyte secretion of IL-8 [22]. Additionally, Morgan et al*.* proposed an organotypic model of the mammary duct to investigate response to the aromatase inhibitor (AI) anastrozole in obese patients compared to those of normal weight [40]. Results showed that MCF-7-derived ducts co-cultured with obese-derived stromal cells exhibited reduced sensitivity to anastrozole compared to lean co-cultures. Interestingly, this difference was not seen in a conventional 2D culture system, highlighting the value of 3D in vitro models.

### Patient-derived organoids

The term “organoid” refers to the ability of cells to self-organise themselves into structures that recapitulate, at least in part, the organ architecture from which they were derived while preserving cell–cell and cell-ECM interactions [62]. Therefore, it is proposed that the organoid TME resembles the original TME more accurately than that of traditional 2D cultures. Generally, organoid systems share the following features: (i) are cultured and embedded within an ECM gel, and (ii) form 3D structures of epithelial cells generated from smaller multicellular units. Organoids are considered an intermediate model between in vitro cancer cell lines and xenografts in terms of tumour heterogeneity [63–66]. Patient-derived tumour organoids (PDTOs) are 3D cell culture systems that are generated in vitro from surgically resected patients’ tumours. They are established by adherent tumoral cells and maintain several features of the primary tumour, including cellular and genetic heterogeneity [67], thus providing a more suitable platform for studying tumour progression, invasion, and drug response.

Organoids provide a similar utility to that of 2D cell lines as they can be passaged and cryopreserved, offering researchers the benefit of a more physiologically relevant model with ease. Establishing an organoid culture system is more costly than cultivating cell lines but less expensive than patient derived xenografts (PDX) [68]. However, culturing organoids often entails complex culture requirements and are not presently available for all cancer types. However, this field is constantly expanding, and many commercial entities now provide organoid lines.

#### Applications of patient-derived organoids

Patient-derived organoids can be grown from mechanically or enzymatically dissociated biopsies, keeping tumour cells within the same TME. Therefore, they represent an attractive alternative to investigate therapeutic compounds for BC. These systems (PDTOs) have been employed to evaluate the response to clinically relevant antitumor therapeutics such as paclitaxel, tamoxifen, trastuzumab, and combinations thereof [69, 70]. Breast cancer organoids have also been used to explore mechanisms underlying tumour cell invasion and metastasis. In 2013, Cheung and colleagues used PDTOs grown in collagen type I matrices to identify characteristics of invasive cancer cells in primary breast tumours [67]. They found that among the major BC subtypes, invasion was induced by cancer cells expressing basal epithelial genes such as cytokeratin-14 and p63 [67].

In 2020, Campaner et al., described a 3D PDTO culture system which recapitulated the histological features of four different subtypes of primary BCs [70]. Primary human epithelial cells self-organised to form complex ductal and lobular morphologies. However, PDTOs derived from a HER2-enriched carcinoma, did not show HER2 staining. In the parental tumour, 50% of cancer cells showed high HER2 staining, thus this model was not capable of maintaining all characteristics of the tumour of origin.

Organoid cultures are mainly epithelial cultures devoid of stromal cells and tumour vasculature. Therefore, further research is required into co-culturing organoids with other cell types to mimic genetic and phenotypic characteristics of the TME. Koledova et al*.,* developed a co-culture system containing murine mammary organoids and CAF spheroids [71], though this has yet to be investigated in human cells.

At present, there are no published reports of organoid models investigating the inflammatory obese microenvironment in BC. Therefore, further studies are required looking at co-culturing organoids with other major cell types of the breast TME such as macrophages and adipocytes to investigate BC associated with obesity.

### Explant cultures

The 3D models described so far mostly require single cell suspensions to be inserted into matrices such as hydrogels. However, structures formed from such cultures may not reflect the native anatomy of BC. An alternative approach may be to remove ductal structures from a patient and culture this 3D in vitro as an explant. Explants retain many physical characteristics of the patient and have been used to more accurately model the breast TME. Patient-derived explants (PDE) have been shown to sustain tissue morphology, viability, and endocrine signalling [72, 73]. Moreover, preliminary data using PDEs show patient-specific responses to immunotherapies [74], suggesting the utility of this platform for drug screening and identifying novel biomarkers that could stratify patients.

However, this culture method is quite inaccessible to most laboratories as this relies on the ability to gain fresh, viable tumour samples, therefore, requiring the input of surgeons and pathologists. Furthermore, explant feasibility is largely based on the integrity of the tumour sample, introducing variability of results. Another major limitation of PDEs is their short culture window, as they are reported to only remain viable for around 72 h.

#### Applications of explant cultures in breast cancer

To model the inflammatory obese microenvironment in BC, previous studies have cultured primary AT as explants to develop co-culture systems. A recent study showed that extracellular vesicles isolated from obese adipose tissue explants, increased the proliferative potential of MCF-7 and MDA-MB-231 cells in vitro [75]. Interestingly, the proliferative effects of extracellular vesicles on MCF-7 cells were ERK/MAPK dependent; whereas, migration of MDA-MB-231 cells was dependent on activation of the PI3K/AKT. Similarly, an earlier study subjected breast tumour and pre-neoplastic cells to conditioned medium collected from AT from healthy individuals or BC patients [76]. Conditioned medium from tumour AT explants increased proliferation of both tumour and non-tumour breast epithelial cells and reduced adhesion of MCF-7 cells.

### Microfluidic tumour-on-a-chip devices

Thus far, none of the model systems considered have incorporated fluid flow, which is a major limitation for the study of BC biology. The vascular networks within the breast TME, supply nutrition and oxygen to the primary tumour, as well as aiding cancer cell invasion into the stroma and capillaries through a process known as intravasation [77]. Microfluidic approaches provide a closed system of 3D organotypic cultures that can allow perfused vasculature to be maintained in real time [78, 79]. Multiple cell types can be incorporated into the system, allowing for multicellular interactions to be examined. Organoids can be embedded in separate compartments of the device and linked with microfluidic channels for nutrient and small molecule exchange, modelling the vascular and lymphatic systems [80, 81].

Microfluidics-based assays may have several advantages for in vitro 3D tumour models compared to static systems: (1) the microfluidic system culture environment is highly reflective of the biochemically dynamic properties displayed by tumour tissues in vivo; (2) these systems enable 3D cultures to be performed using either scaffold-based or scaffold-free approaches; (3) microfluidic platforms are compatible with high throughput cancer drug screening. Such systems have led to advances in our understanding of tumour extravasation, intravasation, and immune recruitment to tumour sites [77, 82]. Utilising microfluidic approaches for drug screening may provide a personalised, preclinical screen to predict efficacy and safety for individual patients.

#### Applications of microfluidic systems in breast cancer

In 2021, Humayun et al*.,* developed an organotypic microfluidic model to investigate cancer-vasculature interactions during cancer extravasation in TNBC [83]. This system employed a tubular endothelial vessel generated from induced pluripotent stem cell-derived endothelial cells within a collagen-fibrinogen matrix. Breast cancer cells were then injected through and cultured along the lumen of the endothelial vessel. This approach identified cancer-vascular crosstalk involving increased levels of secreted factors such as IL-6, IL-8, and MMP-3. However, this model has only looked at one element of the breast TME and future studies should focus on the use of multicellular microfluidic approaches.

Recent advances have been made utilising microfluidic systems to model BC-stroma interactions [82, 84]. Lugo-Cintron et al. developed a multicellular microfluidic organotypic model of BC cells and fibroblasts in collagen or fibronectin-rich matrixes to investigate how different ECM compositions and fibroblast compositions impacted BC migration [84]. The greatest migration was observed when TNBC cells were co-cultured with CAFs, however, culture in a fibronectin-rich matrix also demonstrated increased tumour migration. Interestingly, they show that MMP inhibitors are not effective when CAFs are present, suggesting why preclinical studies based on in vitro 2D cultures have failed. This highlights the potential of such model systems to assist clinical trials.

Berger Fridman and colleagues developed two breast TME models using a high throughput microfluidic system [85]. Their ‘pro-inflammatory’ TME model consisted of BC cells with fibroblasts, M1-like macrophages and activated T cells. In comparison, the second ‘immunosuppressive model’ contained M2-like macrophages and non-activated T cells. Results suggested that macrophages switched from an M1-like to an M2-like phenotype in the pro-inflammatory model, exhibiting increased IL-10 and CD86 expression.

Interestingly, most previous microfluidic studies have focussed on TNBC, using the invasive MDA-MB-231 cell line. The usefulness of such microfluidic models could be further improved by incorporating different cell lines that represent distinct BC subtypes, and utilising patient-derived cancer cells. Furthermore, there are currently no published reports of microfluidic models investigating the inflammatory obese microenvironment in BC, providing an avenue for further investigation.

## Current challenges and future directions

As discussed above, 3D co-culture systems offer the potential to better replicate the breast tissue microenvironment and in vivo physiology than 2D culture or animal models, as they can organise into distinct structures resembling functional units of the breast. Additionally, 3D co-culture models provide a useful tool for recapitulating the stromal-mediated effects on tumour development and drug resistance. Nonetheless, there remains a need for novel models that replicate obesity associated adipose inflammation in BC to investigate tumour-adipose interactions. Furthermore, the inclusion of adipocytes within 3D models of BC has mainly utilised stem cells. The differentiation of stem cells into adipocytes may not provide an appropriate source of adipocytes for recapitulating adipose-tumour interactions within the breast, and further studies are required utilising primary adipose tissue.

A recent study showed that breast cancer cells from obese and lean patients showed significant differences in their gene expression profiles, which may suggest a possible reprogramming of mammary epithelial cells in an obese setting [86]. Furthermore, this study showed that obesity had a diverse impact on the immune landscape and stromal populations of the breast TME. Therefore, further mechanistic studies are required investigating breast cancer behaviour in lean vs obese environments. The use of 3D in vitro co-culture systems may provide a useful tool for such mechanistic studies, where cancer cells can be grown in culture medium containing high glucose or fatty acids to mimic caloric overload. As obesity is a rising global epidemic, it is vital to understand how the TME differs between obese and lean patients to develop personalised therapy approaches to treat these distinct BCs accordingly. Given the prevalence of obesity globally, the need to incorporate preclinical obesity models in cancer investigations is imperative.

## Data Availability

Not applicable.

## Abbreviations

2D: Two-dimensional
3D: Three-dimensional
ADSC: Adipose-derived stem cell
ATMs: Adipose tissue macrophages
BC: Breast cancer
BME: Basement membrane extract
CAA: Cancer-associated adipocyte
CLS: Crown-like structures
DCIS: Ductal carcinoma in situ
ECM: Extracellular matrix
HR: Hormone receptor
IL-6: Interleukin 6
IL-8: Interleukin 8
PDE: Patient derived explant
PDTO: Patient derived tumour organoid
PDX: Patient derived xenografts
PEG: Polyethylene glycol
T2D: Type II diabetes
TME: Tumour microenvironment
TNF-α: Tumour necrosis factor alpha
